# Association of caffeine citrate plus vitamin A/D drops with bronchopulmonary dysplasia and respiratory outcomes in preterm infants born at <32 weeks’ gestation: a 72-h landmark retrospective cohort study

**DOI:** 10.3389/fped.2026.1923440

**Published:** 2026-08-26

**Authors:** Rongying Su, Yaohua Pei

**Affiliations:** 1 Department of Pediatrics, Shijiazhuang People’s Hospital, Shijiazhuang, Hebei, China; 2 Department of Neurology, Shijiazhuang People’s Hospital, Shijiazhuang, Hebei, China

**Keywords:** bronchopulmonary dysplasia, caffeine citrate, landmark analysis, preterm infants born at 32 weeks, vitamin A/D drops

## Abstract

**Objective:**

The aim of this work is to examine whether the early addition of vitamin A/D drops to caffeine citrate was associated with bronchopulmonary dysplasia (BPD) and short-term respiratory outcomes in preterm infants born at <32 weeks’ gestation.

**Methods:**

This single-center, 72-h landmark retrospective cohort study included 126 infants admitted to the hospital between October 2022 and November 2024 with a gestational age <32 weeks and birth weight ≤1.5 kg. Exposure was determined at 72 h as either caffeine alone or caffeine plus vitamin A/D drops. Complete-case analyses were performed. Among the 119 infants who survived to 36 weeks’ postmenstrual age, BPD, longitudinal outcomes, and clinical course were evaluated. Multivariable regression and stabilized inverse probability of treatment weighting (IPTW) were applied.

**Results:**

BPD occurred in 20 of 55 survivors (36.4%) receiving caffeine alone and 11 of 64 (17.2%) receiving combination therapy. After adjustment for gestational age, birth weight, and grade III–IV respiratory distress syndrome, combination therapy was associated with a lower risk of BPD [adjusted odds ratio (aOR), 0.36; 95% confidence interval (CI), 0.14–0.90]. The composite outcome of death or BPD occurred in 25 of 60 infants (41.7%) in the caffeine group and 13 of 66 (19.7%) in the combination group (extended-model aOR, 0.33; 95% CI, 0.14–0.80); IPTW results were consistent. Combination therapy was also associated with more favorable changes in blood gas and inflammatory markers, along with shorter durations of respiratory support and hospitalization. No serious adverse event required permanent treatment discontinuation.

**Conclusion:**

Among infants who survived to 72 h, early initiation of vitamin A/D drops as an adjunct to caffeine was associated with lower risks of BPD and death or BPD, as well as more favorable short-term respiratory outcomes. Non-randomized treatment allocation, complete-case selection, and residual confounding preclude causal inference.

## 1 Introduction

Bronchopulmonary dysplasia (BPD) remains one of the most common and consequential respiratory complications of very preterm birth. Contemporary BPD is not simply a disorder of oxygen toxicity; rather, it is a heterogeneous syndrome characterized by arrested alveolar and pulmonary vascular development in the setting of mechanical ventilation, oxygen exposure, inflammation, infection, and nutritional vulnerability (1–3). Reported rates of BPD and the composite outcome of death or BPD vary substantially according to the study population, BPD definition, and level of neonatal care. Gestational age, birth weight, patent ductus arteriosus (PDA), infection, and respiratory support intensity are consistently reported risk factors (4, 5). Existing early BPD prediction models frequently have a high risk of bias, limited external validation, and suboptimal calibration (6). As survival among extremely preterm infants improves, reducing BPD and its long-term pulmonary and neurodevelopmental burden without increasing mortality remains a major challenge in neonatal intensive care.

Caffeine citrate is the standard pharmacologic therapy for apnea of prematurity. The Caffeine for Apnea of Prematurity (CAP) randomized trial showed that caffeine reduced BPD and the duration of positive-pressure ventilation, while conferring some long-term neurodevelopmental benefit (7, 8). Systematic review evidence also supports reductions in apnea, respiratory support requirements, and selected adverse outcomes (9). However, the response to caffeine varies according to gestational maturity, disease severity, pharmacokinetics, and individual susceptibility (10). Stimulation of respiratory drive alone cannot fully address impaired lung development, oxidative stress, or inflammatory injury. Ventilatory strategies and other lung-protective measures therefore remain important determinants of BPD risk (11).

Vitamin A plays an important role in epithelial differentiation, alveolar formation, and tissue repair. A landmark randomized trial found that intramuscular vitamin A modestly reduced death or chronic lung disease among extremely-low-birth-weight infants (12). Earlier systematic reviews suggested that enteral supplementation might improve selected respiratory outcomes (13). However, neither the multicenter NeoVitaA trial nor an updated 2025 meta-analysis demonstrated a significant reduction in moderate-to-severe BPD or death with enteral vitamin A, suggesting that any effect may depend on formulation, dose, route, and baseline nutritional status (14, 15). Vitamin D is involved in immune regulation, inflammation, and lung development. Low 25-hydroxyvitamin D [25-(OH)D] concentrations have been associated with BPD and respiratory morbidity in preterm infants (16–18), but evidence from intervention studies and systematic reviews of early supplementation remains inconsistent (19–21).

Accordingly, this study used a 72-h postnatal landmark design to evaluate whether early vitamin A/D drops added to standard caffeine therapy were associated with BPD at 36 weeks’ postmenstrual age (PMA), the composite outcome of death or BPD, and short-term respiratory outcomes. Secondary objectives were to compare longitudinal changes in 25-(OH)D, respiratory indices, and inflammatory markers; evaluate clinical course and safety; and address confounding through multivariable models, inverse probability of treatment weighting (IPTW), infection exclusion analyses, and gestational age stratification.

## 2 Materials and methods

### 2.1 Study design and participants

This single-center retrospective cohort study reviewed the medical records of preterm infants admitted to the neonatal department of Shijiazhuang People's Hospital between October 2022 and November 2024. A 72-h postnatal landmark was used to reduce immortal time bias; only infants who were alive and still hospitalized at 72 h were eligible. Infants who had initiated daily vitamin A/D drops orally or through a gastric tube by the landmark were assigned to the combination group, whereas those who had not were assigned to the caffeine group. Exposure status was fixed at 72 h regardless of subsequent treatment interruption, discontinuation, or clinical response. Both strategies were used concurrently, and all infants received caffeine citrate. Initiation of vitamin A/D drops was based on the attending neonatologist's assessment of enteral route feasibility, feeding tolerance, contraindications, product availability, and clinical stability, all documented before the landmark. Treatment allocation was not based on subsequent laboratory changes or study outcomes.

Inclusion criteria were as follows: (1) gestational age <32 weeks; (2) birth weight ≤1.5 kg; (3) admission to the neonatal intensive care unit within 24 h after birth; (4) receipt of invasive or non-invasive respiratory support during hospitalization; (5) treatment with caffeine citrate after birth; (6) survival and continued hospitalization at 72 h, with treatment exposure before the landmark ascertainable; (7) ascertainable survival status before 36 weeks’ PMA and, for survivors, available respiratory support information at 36 weeks’ PMA; (8) availability of the key baseline covariates required for the primary models; and (9) for survivors included in longitudinal analyses, paired pretreatment and day-14 ± 2 laboratory and respiratory measurements.

Exclusion criteria were as follows: (1) major congenital malformation, complex congenital heart disease, chromosomal abnormality, or inherited metabolic disorder; (2) definite contraindication to a study drug; (3) transfer to the hospital after 24 h of age with incomplete perinatal data; (4) death before the 72 h landmark; (5) transfer, discharge against medical advice, or loss to follow-up precluding ascertainment of survival and BPD status at 36 weeks’ PMA; (6) inability to determine exposure to vitamin A/D drops or key baseline covariates before the landmark; and (7) for survivors, absence of the paired measurements required for the longitudinal analyses.

Complete-case analyses included all records that met the clinical and data availability criteria; no *a priori* sample size calculation was performed. Among the 119 survivors, 31 BPD events occurred. To limit overfitting in this small sample, the primary logistic regression model was restricted to the treatment group and three core confounders: gestational age, birth weight, and respiratory distress syndrome (RDS) severity. An extended model that additionally included PDA and early invasive ventilation was used only as a robustness analysis.

The study was approved by the Ethics Committee of Shijiazhuang People's Hospital [Hospital Scientific Research Ethics Review (2021) No. 127]. Because this retrospective study used only de-identified medical record data, the requirement for written informed consent from parents or guardians was waived. The study was conducted in accordance with the Declaration of Helsinki.

### 2.2 Treatment protocols

Both groups received thermal care, airway management, fluid and nutritional support, pulmonary surfactant when indicated, and invasive or non-invasive respiratory support. Respiratory mode, FiO_2_, and ventilator settings were adjusted by the attending physician according to continuous SpO_2_ monitoring, arterial blood gas results, work of breathing, and overall clinical status; the target SpO_2_ range was 90%–95%. Both groups followed the same nutritional pathway for preterm infants. Minimal enteral feeding, availability of human milk, and initiation of parenteral nutrition within 24 h were recorded as pre-landmark nutritional variables. Time to first enteral feeding, time to full enteral feeding, and predominantly human milk feeding during hospitalization were considered post-exposure process measures and were not included in the primary outcome models to avoid overadjustment for potential mediators. Background vitamin intake from human milk, human milk fortifier, preterm formula, and parenteral nutrition was recorded when available but was not converted to cumulative daily doses.

#### 2.2.1 Caffeine citrate group

Caffeine citrate was initiated within 24 h after birth [Feitong; China Resources Double-Crane Limin Pharmaceutical (Jinan) Co., Ltd.; National Medical Products Administration approval No. H20203034; 1 mL:20 mg, equivalent to 10 mg anhydrous caffeine]. A loading dose of 20 mg/kg, calculated as caffeine citrate, was infused intravenously over 30 min, followed 24 h later by a maintenance dose of 5 mg/kg every 24 h. Intravenous administration was used until enteral feeding was established, after which treatment was switched to oral or gastric tube administration when feasible. The maintenance dose could be increased to 10 mg/kg/day for persistent, clinically significant apnea. Caffeine was discontinued at 34–35 weeks’ PMA after 5–7 consecutive days without clinically significant apnea and with stable respiratory status. Heart rate and apnea events were monitored continuously, and feeding tolerance was recorded daily (7).

#### 2.2.2 Caffeine citrate plus vitamin A/D drops group

In addition to the caffeine regimen, vitamin A/D drops were administered as a soft-capsule formulation (Yikexin; Shandong Dyne Marine Biopharmaceutical Co., Ltd.; National Medical Products Administration approval No. H37022973; each capsule containing 1,500 IU vitamin A and 500 IU vitamin D_3_). Treatment was initiated within 72 h after birth at a dose of one capsule once daily. The tip of the capsule was cut, and the contents were administered directly into the mouth or through a gastric tube; after tube administration, the tube was flushed with a small volume of warm water in accordance with the nursing protocol. Treatment continued until 36 weeks’ PMA or hospital discharge, whichever occurred first. Administration was temporarily withheld when enteral dosing was not tolerated and resumed once enteral feeding was reestablished.

Treatment implementation was verified against medication orders and nursing administration records. The timing of the first vitamin A/D dose, planned and actual treatment durations, and the timing and duration of any consecutive treatment interruptions were recorded. Vomiting, reflux, abdominal distension, gastric residuals, feeding interruptions, and heart rate were assessed daily. Serum calcium, phosphorus, alkaline phosphatase (ALP), alanine aminotransferase, aspartate aminotransferase, and creatinine were measured at baseline and weekly thereafter; 25-(OH)D was measured before treatment and on day 14 ± 2. If serum calcium exceeded 2.75 mmol/L (approximately 11.0 mg/dL), 25-(OH)D exceeded 100 ng/mL, sustained heart rate exceeded 180 beats/min, or vitamin A/D toxicity was suspected, the attending physician withheld vitamin A/D drops and repeated the relevant assessment before deciding whether treatment could resume. Infants who did not complete the planned course because of death or early discharge remained in the group assigned at the 72-h landmark.

### 2.3 Data collection and outcome definitions

Two trained investigators independently extracted the data, with discrepancies resolved by a third investigator. Baseline variables included gestational age, birth weight, sex, delivery mode, 1- and 5-min Apgar scores, small-for-gestational-age status, premature rupture of membranes, chorioamnionitis, antenatal corticosteroid completion, surfactant use, RDS severity, PDA within 72 h, initial respiratory support mode, FiO_2_, mean airway pressure (MAP), PaCO_2_, early nutritional variables, and admission period (2022–2023 or 2024). Initial respiratory support was classified as continuous positive airway pressure (CPAP), nasal intermittent positive-pressure ventilation (NIPPV), conventional invasive ventilation, or high-frequency oscillatory ventilation (HFOV), and dichotomized as invasive or non-invasive for regression analyses. Post-landmark process variables included feeding milestones, predominantly human milk feeding, late-onset sepsis, necrotizing enterocolitis (NEC) stage II or higher, pulmonary hemorrhage, postnatal corticosteroid use, and transfusion. Pre-landmark allocation-related variables included enteral route availability, minimal enteral feeding, feeding intolerance, contraindications, product availability, and clinical instability. Feeding intolerance comprised documented vomiting, clinically important abdominal distension, bloody stools, marked gastric residuals, or interruption of enteral feeding; clinical instability comprised escalation of respiratory or circulatory support or another acute major event that led the attending neonatologist to defer supplementation.

RDS was diagnosed on the basis of postnatal clinical presentation, chest imaging, and respiratory support requirements, and was graded I–IV according to chest radiographic findings. Grade III–IV RDS was entered into the regression models as a binary covariate. PDA was defined according to echocardiographic findings and clinical documentation within 72 h after birth to ensure that it was present before or near the exposure landmark.

Initial respiratory support measurements were those obtained closest to 24 h after birth, before the first vitamin A/D dose and after respiratory settings had remained stable for at least 30 min. FiO_2_ was recorded on a scale from 0.21 to 1.00. For CPAP, MAP was the set continuous airway pressure; for NIPPV, conventional invasive ventilation, and HFOV, device-recorded MAP was used. Initial PaCO_2_ was defined as the arterial blood gas value closest to the respiratory support record within the same time window. Because peak inspiratory pressure and positive end-expiratory pressure arose from the same pressure support system as MAP and depended strongly on respiratory mode, they were used only to verify source records and were not included in baseline comparisons or multivariable models, thereby avoiding redundant information and collinearity. Pretreatment laboratory values were the measurements obtained closest to caffeine initiation and within the preceding 24 h; post-treatment values were obtained on treatment day 14 ± 2. Variables included 25-(OH)D, ALP, serum calcium, phosphorus, parathyroid hormone (PTH), C-reactive protein (CRP), interleukin-6 (IL-6), tumor necrosis factor-α (TNF-α), transforming growth factor-β1 (TGF-β1), arterial PaO_2_ and PaCO_2_, and FiO_2_ at the time of blood sampling. Blood gas analyses included only radial arterial or umbilical arterial samples; capillary samples were not combined with arterial samples. Respiratory mode and FiO_2_ remained stable for at least 30 min before sampling, and no suctioning, intubation, or other acute procedure was performed during the preceding 30 min. SpO_2_, respiratory mode, and FiO_2_ were recorded concurrently. Longitudinal analyses included the 119 survivors who remained hospitalized at the day-14 assessment and had paired arterial blood gas and laboratory measurements within the prespecified windows.

Serum 25-(OH)D and PTH were measured by electrochemiluminescence immunoassay. ALP was measured using a kinetic colorimetric method, serum calcium by the o-cresolphthalein complexone colorimetric method, phosphorus by the phosphomolybdate ultraviolet method, and CRP by immunoturbidimetry on automated laboratory analyzers. Serum IL-6, TNF-α, and TGF-β1 were quantified by enzyme-linked immunosorbent assay using commercially available kits. Arterial PaO_2_ and PaCO_2_ were measured immediately after sampling using an automated blood gas analyzer. All assays were performed in the hospital central laboratory according to routine standard operating procedures and internal quality control requirements. Samples were processed and stored in accordance with the corresponding laboratory protocols.

Infection at the post-treatment sampling time point was defined as a positive blood culture, antimicrobial treatment for suspected or confirmed sepsis, or another definite infection, such as NEC, within 48 h before or after blood sampling. The primary inflammatory marker models additionally adjusted for maternal chorioamnionitis and infection status at sampling, and a sensitivity analysis excluded infants with infection.

The primary outcome was BPD at 36 weeks’ PMA. BPD severity was classified according to the Jensen criteria (1): no respiratory support, no BPD; nasal cannula flow ≤2 L/min, grade 1 BPD; nasal cannula flow >2 L/min or non-invasive positive-pressure support (e.g., CPAP or NIPPV), grade 2 BPD; and ongoing invasive mechanical ventilation, grade 3 BPD. Infants who remained hospitalized were assessed at the bedside at 36 weeks’ PMA (within ±3 days). For infants discharged earlier, respiratory support status during the same window was determined from outpatient records, home oxygen prescriptions, device flow settings, and structured telephone follow-up. Two neonatologists who were unaware of treatment assignment independently adjudicated BPD; disagreements were resolved by a third neonatologist. Death before 36 weeks’ PMA was defined as all-cause death between the 72-h landmark and 36 weeks’ PMA. The composite outcome comprised death before 36 weeks’ PMA or BPD among infants who survived to 36 weeks, with each infant counted once.

Secondary outcomes included the durations of invasive mechanical ventilation, oxygen dependence, and hospitalization; age at first enteral feeding; time to full enteral feeding; predominantly human milk feeding during hospitalization; and treatment-related adverse events. Duration of invasive mechanical ventilation was calculated from the first intubation to successful extubation, defined as no reintubation within 48 h. Oxygen dependence was measured from the first time FiO_2_ >0.21 was required until the target SpO_2_ was maintained in room air for 24 consecutive hours; home oxygen use after discharge was included. Length of stay was calculated only among infants discharged alive. Feeding intolerance was defined as suspension or reduction of enteral feeding for ≥24 h because of abdominal distension, gastric residuals, vomiting, or bloody stools. Tachycardia, hypercalcemia, liver function abnormalities, 25-(OH)D > 100 ng/mL, and suspected vitamin A/D toxicity were adjudicated according to prespecified thresholds. The safety observation window began at the 72-h landmark and ended at discontinuation of the last study regimen medication, discharge, death, or 36 weeks’ PMA, whichever occurred first. All 126 infants in the landmark cohort were included in the safety analysis.

### 2.4 Missing data

**Figure 1:**
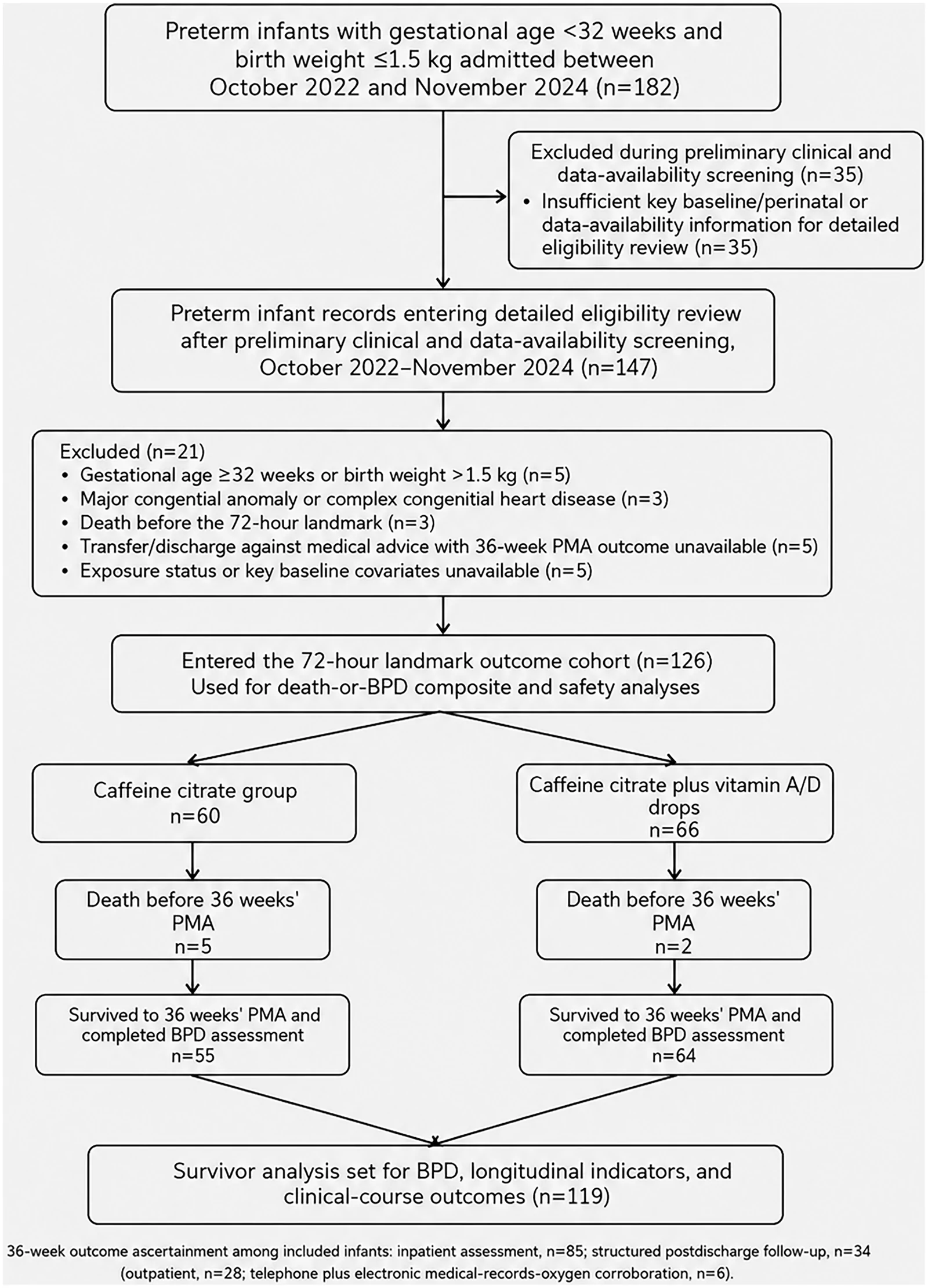
Participant flow and analysis sets. Of 182 potentially eligible preterm infants screened, 147 records underwent detailed eligibility review and 126 infants entered the 72-h landmark cohort (60 in the caffeine citrate group and 66 in the caffeine citrate plus vitamin A/D drops group). This cohort was used for analyses of death or BPD and safety. A total of 119 infants survived to 36 weeks’ PMA and completed BPD assessment (55 and 64, respectively), forming the survivor analysis set for BPD, longitudinal indicators, and clinical course outcomes. Outcomes at 36 weeks’ PMA were ascertained during hospitalization in 85 infants and after discharge in 34 infants.

Participant flow is shown in Figure 1. Records lacking sufficient data to determine eligibility, exposure, key baseline covariates, or 36-week outcomes were excluded during screening. Complete-case analyses were performed without statistical imputation. The 126-infant landmark cohort had complete exposure, covariate, outcome, and safety data. All 119 survivors included in longitudinal analyses had paired pretreatment and day-14 ± 2 measurements. Outcomes were ascertained for all 34 infants discharged before 36 weeks’ PMA through structured follow-up.

### 2.5 Statistical analysis

Statistical analyses were performed using SPSS version 26.0 and R version 4.3.2. In R, the lme4 and lmerTest packages were used for linear mixed-effects models; logistf for Firth-corrected logistic regression; MASS for ordinal logistic regression; WeightIt, cobalt, and survey for propensity score weighting, balance diagnostics, and weighted effect estimation; sandwich and lmtest for heteroscedasticity-robust standard errors; car for variance inflation factor (VIF) diagnostics; and the p.adjust function in the stats package for multiple-comparison correction. All tests were two-sided, and *P* < 0.05 was considered statistically significant. Normally distributed continuous variables are presented as mean ± standard deviation, skewed variables as median (25th, 75th percentile), and categorical variables as number (percentage). Reporting followed the STROBE checklist for cohort studies (22).

Baseline continuous variables were compared using the independent-samples t test or Mann–Whitney U test, as appropriate. Categorical variables were compared using the chi-square test or Fisher’s exact test, and ordinal variables using the Wilcoxon rank-sum test. Standardized mean differences (SMDs) were also calculated, with an absolute SMD <0.10 indicating good balance. Balance for the multi-category respiratory support variable was evaluated using Cramér's V. Correlations and VIFs were examined before entering initial invasive support, FiO_2_, MAP, and PaCO_2_ into the same model; a VIF <5 indicated no important multicollinearity. Treatment allocation variables are summarized descriptively in Supplementary Table S4 using numbers, percentages, exact *P*-values, and absolute SMDs. Because these variables were components of routine clinical treatment selection rather than randomly distributed baseline characteristics, SMDs were used primarily to quantify the direction and magnitude of allocation-related imbalance. As a supplementary robustness analysis, the propensity score model was expanded to include enteral route availability by 72 h, pre-landmark feeding intolerance, and documented product availability in addition to the prespecified covariates. Stabilized weights were calculated and truncated at the 1st and 99th percentiles using the same procedure as in the primary IPTW analysis. The untruncated and truncated weight ranges, weighted effective sample size, maximum post-weighting absolute SMD, and weighted outcome estimates are reported in Supplementary Table S5.

For repeated measurements, individual change was calculated as post-treatment minus pretreatment. The primary analysis used linear mixed-effects models with treatment group, time, the group-by-time interaction, and prespecified confounders as fixed effects, and a random intercept for each infant. Models for 25-(OH)D and bone metabolism markers were adjusted for gestational age and birth weight; respiratory marker models were additionally adjusted for RDS severity; and inflammatory marker models were additionally adjusted for chorioamnionitis and infection status at sampling. The group-by-time interaction represented the between-group difference in change. Adjusted between-group differences in change, 95% confidence interval (CIs), and *P-*values were reported.

BPD incidence was analyzed in the 119-infant survivor analysis set using multivariable logistic regression. The primary model adjusted for gestational age, birth weight, and RDS severity; the extended model additionally included PDA diagnosed within 72 h and invasive ventilation within 24 h. Because only 16 grade 2–3 BPD events occurred, this outcome was designated exploratory and analyzed using Firth-corrected logistic regression adjusted only for gestational age, birth weight, and RDS severity; no extended model was fitted. The four-level BPD outcome was assessed separately using exploratory ordinal logistic regression. Because late-onset sepsis occurred after the exposure landmark and could lie on the causal pathway between treatment and BPD, it was excluded from the primary models and added only in a supplementary sensitivity model.

In the 126-infant landmark cohort, the composite outcome of death or BPD was analyzed using logistic regression with the same adjustment sets as the BPD models. To further address measured baseline confounding, a binary logistic regression model estimated the propensity to receive combination therapy. Covariates were gestational age, birth weight, RDS severity, PDA within 72 h, chorioamnionitis, initial respiratory support (invasive vs. non-invasive), initial FiO_2_, initial MAP, initial PaCO_2_, minimal enteral feeding within 24 h, availability of human milk within 24 h, and admission period. Initiation of parenteral nutrition within 24 h was described but not included because its prevalence exceeded 95% in both groups and variability was minimal. Peak inspiratory pressure and positive end-expiratory pressure were not included together with MAP and support mode because of strong structural correlation. Stabilized inverse probability of treatment weights were calculated and truncated below the 1st and above the 99th percentiles. Weighted odds ratios and 95% CIs were estimated using robust sandwich standard errors. Balance was defined as an absolute weighted SMD <0.10; the weight range and effective weighted sample size were reported.

Among survivors, the durations of invasive ventilation, oxygen dependence, and hospitalization, as well as age at first enteral feeding and time to full enteral feeding, were analyzed using multivariable linear regression after residual diagnostics, with adjustment for gestational age, birth weight, and RDS severity, along with heteroscedasticity-robust standard errors. Predominantly human milk feeding during hospitalization was analyzed using multivariable logistic regression with the same adjustments. Late-onset sepsis, NEC stage II or higher, pulmonary hemorrhage, postnatal corticosteroid use, and transfusion were treated as post-landmark in-hospital process variables rather than baseline characteristics. Because some events were infrequent, these outcomes were analyzed using Firth-corrected logistic regression adjusted for gestational age, birth weight, and RDS severity. Safety outcomes were compared in the 72-h landmark cohort using Fisher’s exact tests. For each prespecified safety outcome, the absolute risk difference (combination minus caffeine), its 95% CI calculated using the Newcombe method based on Wilson score intervals, and the corresponding *P*-value were reported.

Sensitivity analyses included the following: (1) exclusion of infants with infection within 48 h before or after post-treatment inflammatory marker sampling; (2) analyses of PaO_2_, PaCO_2_, and FiO_2_ restricted to infants still receiving respiratory support on day 14; (3) addition of late-onset sepsis to the primary BPD and composite outcome models; (4) IPTW analyses of BPD and death or BPD; and (5) stratification by gestational age <28 versus 28 to <32 weeks with an interaction test. Because of the limited sample, infants born at <25 weeks were described only for death, BPD, and the composite outcome without unstable regression estimates.

The 25-(OH)D and bone metabolism markers, respiratory markers, and inflammatory markers were treated as three outcome domains defined before analysis. Within each domain, the Benjamini–Hochberg procedure was used to control the false discovery rate (FDR), and both raw and FDR-adjusted *P-*values were reported. Only 25-(OH)D is presented in the main text; ALP, calcium, phosphorus, and PTH are reported in Supplementary Table S1. All five markers were included in the multiple-comparison correction for the bone metabolism domain. Values <0.001 are reported as *P* < 0.001.

## 3 Results

### 3.1 Participant selection, treatment implementation, and baseline characteristics

Of 182 potentially eligible infants screened, 147 underwent detailed eligibility review, and 126 entered the 72-h landmark cohort after exclusions (60 in the caffeine group and 66 in the combination group). Seven infants died before 36 weeks’ PMA (five in the caffeine group and two in the combination group), leaving 119 survivors who completed BPD assessment and had paired longitudinal measurements (55 and 64, respectively). Outcomes at 36 weeks’ PMA were assessed during hospitalization in 85 infants and after discharge in 34 infants. Figure 1 shows participant flow and the analysis sets.

Caffeine dosing followed the protocol in both groups; the maintenance dose was increased to 10 mg/kg/day in 9 of 60 infants (15.0%) in the caffeine group and 8 of 66 (12.1%) in the combination group (*P* = 0.637). In the combination group, vitamin A/D drops were initiated at a median of 61 h after birth (25th, 75th percentile: 55, 66) and administered for 42.8 ± 12.7 days. Four infants had brief treatment interruptions (three for feeding intolerance and one for hypercalcemia), after which treatment resumed; no permanent discontinuation or crossover occurred.

Pre-landmark allocation-related characteristics are summarized in Supplementary Table S4. None of these characteristics differed significantly between groups, although SMDs indicated modest imbalance in feeding initiation, feeding intolerance, contraindications, and product availability.

Early respiratory support intensity was similar between groups (Cramér's V for support mode: 0.035; absolute SMDs for FiO_2_, MAP, and PaCO_2_: 0.081–0.131), and VIFs of 1.05–1.91 indicated no important multicollinearity. Mild imbalance remained for several baseline variables (absolute SMDs: 0.107–0.172), supporting multivariable adjustment and IPTW (Table 1).

**Table 1:** Baseline characteristics of the 72-h landmark cohort.

Variable	Caffeine group (*n* = 60)	Combination group (*n* = 66)	*P*-value	Absolute SMD/Cramér's V
Gestational age (weeks)	28.38 ± 2.15	28.73 ± 1.96	0.338	0.172
Birth weight (kg)	1.12 ± 0.25	1.16 ± 0.22	0.442	0.138
Male sex	28 (46.7)	35 (53.0)	0.476	0.128
Cesarean delivery	25 (41.7)	31 (47.0)	0.550	0.107
1-min Apgar score	5.58 ± 0.84	5.72 ± 0.98	0.388	0.154
5-min Apgar score	6.40 ± 0.97	6.55 ± 1.02	0.409	0.148
Small for gestational age	11 (18.3)	14 (21.2)	0.686	0.072
Premature rupture of membranes	19 (31.7)	23 (34.8)	0.705	0.068
Chorioamnionitis	7 (11.7)	9 (13.6)	0.740	0.059
Complete antenatal corticosteroid course	41 (68.3)	47 (71.2)	0.725	0.063
Surfactant use	52 (86.7)	57 (86.4)	0.960	0.009
Grade III–IV RDS	31 (51.7)	34 (51.5)	0.986	0.003
PDA diagnosed ≤72 h	20 (33.3)	25 (37.9)	0.595	0.095
Initial respiratory support mode			0.985	0.035
CPAP	7 (11.7)	9 (13.6)	—	—
NIPPV	5 (8.3)	6 (9.1)	—	—
Conventional invasive ventilation	44 (73.3)	47 (71.2)	—	—
High-frequency oscillatory ventilation	4 (6.7)	4 (6.1)	—	—
Initial FiO_2_	0.522 ± 0.060	0.514 ± 0.062	0.464	0.131
Initial MAP (cmH_2_O)	8.62 ± 1.76	8.48 ± 1.69	0.650	0.081
Initial PaCO_2_ (mmHg)	57.9 ± 8.0	57.2 ± 7.5	0.613	0.090
Minimal enteral feeding within 24 h	38 (63.3)	44 (66.7)	0.695	0.070
Human milk available within 24 h	35 (58.3)	41 (62.1)	0.664	0.077
Parenteral nutrition started within 24 h	57 (95.0)	63 (95.5)	1.000	0.021
Admission in 2024	26 (43.3)	31 (47.0)	0.682	0.073

SMD, standardized mean difference; RDS, respiratory distress syndrome; PDA, patent ductus arteriosus; CPAP, continuous positive airway pressure; NIPPV, nasal intermittent positive-pressure ventilation; FiO_2_, fraction of inspired oxygen; MAP, mean airway pressure; PaCO_2_, arterial partial pressure of carbon dioxide; NEC, necrotizing enterocolitis.

The overall chi-square test was used for initial respiratory support mode, and the balance is reported using Cramér's V; absolute SMDs are reported for all other variables. Respiratory support was dichotomized as invasive or non-invasive for multivariable analyses. Late-onset sepsis, NEC, pulmonary hemorrhage, postnatal corticosteroid use, and transfusion occurred after the 72-h landmark and are reported in Table 6.

### 3.2 Change in 25-(OH)D

The increase in 25-(OH)D was greater in the combination group, with an adjusted between-group difference in change of 6.38 ng/mL (95% CI, 5.71–7.05). The result remained significant after FDR correction (Table 2). Other bone metabolism markers are reported in Supplementary Table S1.

**Table 2:** Changes in 25-(OH)D before and after treatment (survivor analysis set, n = 119).

Variable	Caffeine, pre	Caffeine, post	Combination, pre	Combination, post	Adjusted difference in change (95% CI)	Raw *P*	FDR-adjusted *P*
25-(OH)D (ng/mL)	13.22 ± 2.48	13.52 ± 3.00	13.43 ± 2.60	20.19 ± 3.06	6.38 (5.71–7.05)	<0.001	<0.001

25-(OH)D, 25-hydroxyvitamin D; CI, confidence interval; FDR, false discovery rate.

Values are mean ± standard deviation. The adjusted difference in change represents the group-by-time interaction from a linear mixed-effects model with a random intercept for each infant, adjusted for gestational age and birth weight. Raw and Benjamini–Hochberg FDR-adjusted *P*-values are reported.

### 3.3 Respiratory indices

By treatment day 14 ± 2, PaO_2_ had increased and PaCO_2_ and FiO_2_ had decreased in both groups, with more favorable changes in the combination group. Adjusted between-group differences in change were 4.08 mmHg, −2.69 mmHg, and −0.028, respectively (Table 3). At the time of sampling, 94 infants remained on respiratory support and 25 were breathing room air.

**Table 3:** Changes in respiratory indices before and after treatment (survivor analysis set, *n* = 119).

Variable	Caffeine, pre	Caffeine, post	Combination, pre	Combination, post	Adjusted difference in change (95% CI)	Raw P	FDR-adjusted P
PaO_2_ (mmHg)	48.32 ± 6.97	59.63 ± 8.69	48.44 ± 5.96	63.77 ± 6.72	4.08 (2.44 to 5.72)	<0.001	<0.001
PaCO_2_ (mmHg)	57.55 ± 7.90	51.47 ± 8.16	56.92 ± 7.39	48.12 ± 8.38	−2.69 (−4.21 to −1.18)	<0.001	<0.001
FiO_2_	0.523 ± 0.059	0.330 ± 0.065	0.513 ± 0.063	0.291 ± 0.060	−0.028 (−0.054 to −0.001)	0.039	0.039

PaO_2_, arterial partial pressure of oxygen; PaCO_2_, arterial partial pressure of carbon dioxide; FiO_2_, fraction of inspired oxygen; RDS, respiratory distress syndrome; CI, confidence interval; FDR, false discovery rate.

Values are mean ± standard deviation. The adjusted difference in change represents the group-by-time interaction from a linear mixed-effects model with a random intercept for each infant, adjusted for gestational age, birth weight, and RDS severity. Raw and Benjamini–Hochberg FDR-adjusted *P*-values are reported.

### 3.4 Inflammatory markers

Adjusted reductions in CRP, IL-6, TNF-α, and TGF-β1 were greater in the combination group, and all remained significant after FDR correction (Table 4).

**Table 4:** Changes in inflammatory markers before and after treatment (survivor analysis set, *n* = 119).

Variable	Caffeine, pre	Caffeine, post	Combination, pre	Combination, post	Adjusted difference in change (95% CI)	Raw *P*	FDR-adjusted *P*
CRP (mg/L)	20.57 ± 4.35	17.11 ± 4.95	22.41 ± 4.78	16.24 ± 5.53	−2.64 (−3.52 to −1.75)	<0.001	<0.001
IL-6 (pg/mL)	35.53 ± 5.76	29.10 ± 6.60	35.10 ± 5.05	25.84 ± 6.03	−2.79 (−3.88 to −1.70)	<0.001	<0.001
TNF-α (pg/mL)	39.88 ± 6.54	36.15 ± 7.14	40.43 ± 6.56	31.67 ± 6.64	−5.06 (−6.37 to −3.75)	<0.001	<0.001
TGF-*β*1 (pg/mL)	584.05 ± 61.99	459.69 ± 78.73	601.22 ± 65.44	408.09 ± 73.99	−68.45 (−85.00 to −51.90)	<0.001	<0.001

CRP, C-reactive protein; IL-6, interleukin-6; TNF-α, tumor necrosis factor-α; TGF-β1, transforming growth factor-β1; CI, confidence interval; FDR, false discovery rate.

Values are mean ± standard deviation. The adjusted difference in change represents the group-by-time interaction from a linear mixed-effects model with a random intercept for each infant, adjusted for gestational age, birth weight, maternal chorioamnionitis, and infection status at sampling. Raw and Benjamini–Hochberg FDR-adjusted *P*-values are reported.

### 3.5 BPD, death, and composite outcomes

Among the 119 survivors, BPD occurred in 20 of 55 infants (36.4%) in the caffeine group and 11 of 64 (17.2%) in the combination group. According to the Jensen criteria, BPD grades 1, 2, and 3 occurred in 9, 7, and 4 infants in the caffeine group and 6, 3, and 2 infants in the combination group, respectively. Grade 2–3 BPD occurred in 11 of 55 infants (20.0%) in the caffeine group and 5 of 64 (7.8%) in the combination group and was analyzed only as an exploratory outcome. After adjustment for gestational age, birth weight, and RDS severity, combination therapy was associated with a lower risk of BPD [adjusted odds ratio (aOR), 0.36; 95% CI, 0.14–0.90; *P* = 0.029]. After additional adjustment for PDA within 72 h and early invasive ventilation, the aOR was 0.38 (95% CI, 0.15–0.95; *P* = 0.038). In the 126-infant landmark cohort, death or BPD occurred in 25 of 60 infants (41.7%) in the caffeine group and 13 of 66 (19.7%) in the combination group. The aORs were 0.31 (95% CI, 0.13–0.74) in the primary model and 0.33 (95% CI, 0.14–0.80) in the extended model (Table 5).

**Table 5:** Primary and exploratory BPD, mortality, and composite outcomes.

Outcome	Caffeine group	Combination group	Unadjusted OR (95% CI)	Unadjusted *P*	Primary-model aOR (95% CI)	*P*-value	Extended-model aOR (95% CI)	*P*-value
Death before 36 weeks’ PMA	5/60 (8.3)	2/66 (3.0)	0.34 (0.06–1.84)	0.213	—	—	—	—
BPD	20/55 (36.4)	11/64 (17.2)	0.36 (0.16–0.85)	0.020	0.36 (0.14–0.90)	0.029	0.38 (0.15–0.95)	0.038
Grade 2–3 BPD (exploratory)	11/55 (20.0)	5/64 (7.8)	0.34 (0.11–1.05)	0.060	0.40 (0.13–1.27)	0.121	—	—
Death or BPD	25/60 (41.7)	13/66 (19.7)	0.34 (0.16–0.76)	0.008	0.31 (0.13–0.74)	0.008	0.33 (0.14–0.80)	0.014

BPD, bronchopulmonary dysplasia; PMA, postmenstrual age; OR, odds ratio; aOR, adjusted odds ratio; CI, confidence interval; RDS, respiratory distress syndrome; PDA, patent ductus arteriosus.

The primary model adjusted for gestational age, birth weight, and RDS severity. The extended model additionally adjusted for PDA diagnosed within 72 h and invasive mechanical ventilation within 24 h. Each infant was counted once in the death-or-BPD composite. Because grade 2–3 BPD events were infrequent, this exploratory outcome was analyzed using Firth-corrected logistic regression adjusted only for gestational age, birth weight, and RDS severity; no extended model was fitted. The exploratory aOR was 0.40 (95% CI, 0.13–1.27; *P* = 0.121). Ordinal logistic regression of BPD grade was also exploratory; the adjusted common OR was 0.43 (95% CI, 0.18–1.04; *P* = 0.063) and was not used for primary inference.

### 3.6 Clinical course and nutritional support outcomes

Clinical course and nutritional support outcomes were analyzed among the 119 survivors, whereas late-onset sepsis, NEC stage II or higher, pulmonary hemorrhage, postnatal corticosteroid use, and transfusion were analyzed in the full 126-infant landmark cohort. After adjustment for gestational age, birth weight, and RDS severity, the combination group had 3.2 fewer days of invasive ventilation, 8.0 fewer days of oxygen dependence, and 7.8 fewer days of hospitalization. Between-group differences in age at first enteral feeding, time to full enteral feeding, predominantly human milk feeding during hospitalization, and the specified in-hospital events were not statistically significant (Table 6).

**Table 6:** Clinical course and nutritional support outcomes among survivors and in-hospital events in the landmark cohort.

Variable	Caffeine group	Combination group	Adjusted effect (95% CI)	*P*-value
Survivor analysis set (*n* = 119; caffeine *n* = 55, combination *n* = 64)				
Invasive ventilation (days)	10.9 ± 6.4	7.4 ± 5.4	−3.2 (−5.2 to −1.2)	0.001
Oxygen dependence (days)	43.4 ± 14.1	34.7 ± 12.2	−8.0 (−11.8 to −4.1)	<0.001
Hospital stay (days)	61.5 ± 13.5	52.9 ± 13.6	−7.8 (−12.0 to −3.7)	<0.001
First enteral feeding (days)	2.3 ± 0.6	2.1 ± 0.6	−0.2 (−0.4 to 0.1)	0.145
Full enteral feeding (days)	16.0 ± 3.1	15.5 ± 3.3	−0.3 (−1.3 to 0.7)	0.568
Predominantly human milk feeding	31 (56.4)	39 (60.9)	1.17 (0.56 to 2.44)	0.686
72 h landmark cohort (*n* = 126; caffeine *n* = 60, combination *n* = 66)				
Late-onset sepsis	11/60 (18.3)	10/66 (15.2)	0.82 (0.31 to 2.19)	0.693
NEC stage II or higher	5/60 (8.3)	4/66 (6.1)	0.76 (0.19 to 3.09)	0.704
Pulmonary hemorrhage	4/60 (6.7)	3/66 (4.5)	0.69 (0.14 to 3.42)	0.653
Postnatal corticosteroids	8/60 (13.3)	7/66 (10.6)	0.79 (0.26 to 2.41)	0.681
Transfusion	29/60 (48.3)	28/66 (42.4)	0.86 (0.42 to 1.77)	0.680

aOR, adjusted odds ratio; CI, confidence interval; RDS, respiratory distress syndrome; NEC, necrotizing enterocolitis.

Adjusted effects for continuous outcomes are between-group mean differences; the effect for predominantly human milk feeding is a conventional logistic regression aOR; and the effects for in-hospital events are Firth-corrected aORs. All models adjusted for gestational age, birth weight, and RDS severity.

### 3.7 Safety

Adverse events were predominantly mild, and no serious adverse event required permanent treatment discontinuation. Absolute risk differences were small, and all 95% CIs included zero (Table 7). One infant in the combination group developed transient hypercalcemia. No treatment-related liver function abnormality, 25-(OH)D > 100 ng/mL, or suspected vitamin A/D toxicity occurred.

**Table 7:** Adverse events in the 72-h landmark cohort.

Adverse event	Caffeine (*n* = 60)	Combination (*n* = 66)	Risk difference, % (95% CI)	*P*-value
Tachycardia	4 (6.7)	5 (7.6)	0.9 (−9.3 to 10.7)	1.000
Feeding intolerance	6 (10.0)	8 (12.1)	2.1 (−9.6 to 13.5)	0.704
Hypercalcemia	0	1 (1.5)	1.5 (−4.6 to 8.1)	1.000
25-(OH)D > 100 ng/mL	0	0	0.0 (−6.0 to 5.5)	1.000
Permanent discontinuation due to an adverse event	0	0	0.0 (−6.0 to 5.5)	1.000
Liver function abnormality	0	0	0.0 (−6.0 to 5.5)	1.000
Suspected vitamin A/D toxicity	0	0	0.0 (−6.0 to 5.5)	1.000

CI, confidence interval; 25-(OH)D, 25-hydroxyvitamin D.

Risk difference was calculated as the event proportion in the combination group minus that in the caffeine group. The 95% CIs were calculated using the Newcombe method based on Wilson score intervals. Two-sided *P*-values were obtained using Fisher’s exact tests and are reported for every prespecified safety outcome, including outcomes with no events in either group.

### 3.8 Sensitivity and subgroup analyses

Fifteen infants had an infection within 48 h before or after post-treatment inflammatory marker sampling (eight in the caffeine group and seven in the combination group). After excluding these infants, 104 remained. The adjusted between-group differences in change were −2.79 mg/L (95% CI, −3.77 to −1.81) for CRP, −2.69 pg/mL (95% CI, −3.88 to −1.49) for IL-6, −5.38 pg/mL (95% CI, −6.79 to −3.96) for TNF-α, and −67.44 pg/mL (95% CI, −86.04 to −48.84) for TGF-*β*1, consistent with the primary analyses.

Among infants still receiving respiratory support on day 14, adjusted between-group differences in change were 4.46 mmHg for PaO_2_ (95% CI, 2.68–6.24; *P* < 0.001), −2.88 mmHg for PaCO_2_ (95% CI, −4.51 to −1.25; *P* < 0.001), and −0.033 for FiO_2_ (95% CI, −0.061 to −0.005; *P* = 0.022), consistent with the primary analyses.

After minimal enteral feeding and availability of human milk within 24 h were added to the propensity score model, untruncated stabilized IPTW weights ranged from 0.53 to 2.79; after truncation at the 1st and 99th percentiles, the truncated range was 0.62–2.34. The weighted effective sample size was 119.8. After weighting, the absolute SMDs for all covariates, including initial respiratory support, FiO_2_, MAP, PaCO_2_, and the two pre-landmark nutritional variables, were <0.05, with a maximum absolute SMD of 0.041 (Supplementary Table S2). IPTW analyses showed that combination therapy was associated with lower risks of BPD (weighted OR, 0.44; 95% CI, 0.19–0.99; *P* = 0.047) and death or BPD (weighted OR, 0.41; 95% CI, 0.19–0.91; *P* = 0.029). When late-onset sepsis was added to the extended models, the aOR was 0.40 for BPD (95% CI, 0.16–1.02; *P* = 0.055) and 0.34 for death or BPD (95% CI, 0.14–0.82; *P* = 0.016); the direction of effect was unchanged. In the expanded propensity score sensitivity analysis that additionally included enteral route availability by 72 h, pre-landmark feeding intolerance, and documented product availability, untruncated stabilized weights ranged from 0.49 to 3.08 and truncated weights from 0.59 to 2.48. The weighted effective sample size was 117.6, and the maximum post-weighting absolute SMD was 0.047, indicating satisfactory balance across the expanded covariate set. The weighted OR was 0.46 (95% CI, 0.20–1.06; *P* = 0.068) for BPD and 0.43 (95% CI, 0.19–0.96; *P* = 0.040) for death or BPD (Supplementary Table S5). These estimates were treated as sensitivity results because the allocation-related variables could not be fully separated from the clinical decision to initiate supplementation.

**Figure 2:**
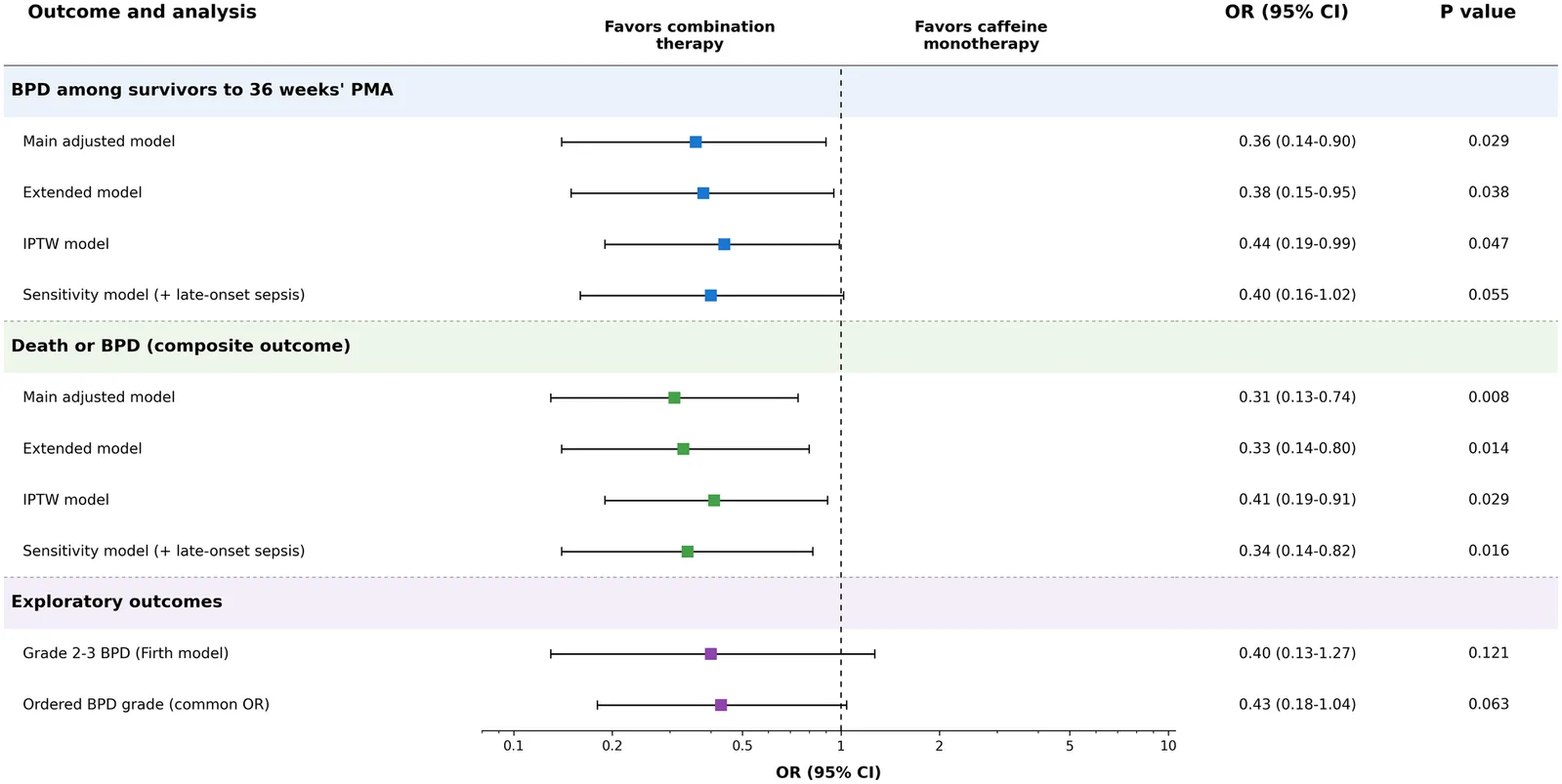
Forest plot of the main and exploratory outcome estimates. Squares indicate odds ratios, horizontal lines indicate 95% confidence intervals, and the vertical dashed line denotes an odds ratio of 1. Main models adjusted for gestational age, birth weight, and grade III–IV respiratory distress syndrome. Extended models additionally adjusted for patent ductus arteriosus diagnosed within 72 h and invasive ventilation within 24 h. IPTW estimates were obtained using stabilized inverse probability of treatment weighting. Sensitivity models additionally included late-onset sepsis. Analyses of grade 2–3 BPD and ordered BPD grade were exploratory.

After stratification by gestational age (<28 vs. 28 to <32 weeks), the treatment-by-gestational-age interaction was not significant (*P* = 0.238). Fourteen infants were born at <25 weeks: eight in the caffeine group and six in the combination group. Death before 36 weeks’ PMA occurred in two of eight and one of six infants, respectively; BPD among survivors occurred in five of six and three of five; and death or BPD occurred in seven of eight and four of six. Given the small sample, these findings are descriptive only (Supplementary Table S3). Figure 2 summarizes the main, extended, IPTW, late-onset sepsis sensitivity, and exploratory effect estimates.

## 4 Discussion

Using a 72-h postnatal landmark design, this study found that among infants born at <32 weeks’ gestation who received standard caffeine therapy and survived to the landmark, the early addition of vitamin A/D drops was associated with a lower risk of BPD among survivors and a lower risk of the composite outcome of death or BPD in the landmark cohort. The combination group also showed more favorable changes in PaO_2_, PaCO_2_, FiO_2_, and inflammatory markers on treatment day 14, along with shorter durations of invasive ventilation, oxygen dependence, and hospitalization. No increase in serious adverse events was observed. Findings were generally consistent across multivariable regression, IPTW, infection exclusion analyses, blood gas analyses restricted to infants who remained on respiratory support, and gestational age stratification. Analyses of grade 2–3 BPD and BPD severity were exploratory because of the limited number of events. Importantly, this non-randomized retrospective cohort study identifies associations rather than definitive treatment effects.

The evidence supporting caffeine is substantial. In addition to the CAP trial, a systematic review of methylxanthines found that caffeine reduces apnea, facilitates weaning from ventilation, and may lower the risk of BPD (23). Proposed mechanisms include adenosine receptor antagonism, increased responsiveness of the respiratory center to carbon dioxide, improved diaphragmatic contractility, and reduced intermittent hypoxemia. Nevertheless, the response to caffeine varies substantially among infants and may depend on maturity, caffeine exposure, and genetic background (24). The differences observed in the combination group therefore cannot be attributed entirely to vitamin supplementation, and unmeasured differences in caffeine dosing or respiratory management cannot be excluded.

Vitamin A has a well-established biological role in lung development. Its metabolite—retinoic acid—participates in epithelial differentiation, alveolar formation, and tissue repair. The landmark randomized trial used 5,000 IU intramuscularly three times weekly for 4 weeks and observed a modest reduction in death or chronic lung disease (12). However, findings have differed by formulation and route; neither the NeoVitaA trial nor the updated meta-analysis found that enteral vitamin A significantly reduced moderate-to-severe BPD or death (14, 15). The fixed-dose oral combination used in the present study provided 1,500 IU vitamin A and 500 IU vitamin D_3_ daily, which differed in dose and route from the classic intramuscular regimen and high-dose enteral trials. Accordingly, the observed association cannot be attributed specifically to vitamin A or considered equivalent to findings from trials of vitamin A alone.

The vitamin D findings require particularly cautious interpretation. Observational studies and meta-analyses have associated low 25-(OH)D concentrations with BPD or respiratory morbidity in preterm infants (16–18), whereas some small intervention studies, systematic reviews, and network meta-analyses have suggested a possible benefit of early supplementation (19–21). However, vitamin D studies vary widely in dose, timing, baseline vitamin D status, and outcome definitions. The combined product used in this study provided 500 IU vitamin D_3_ daily together with vitamin A. In addition, vitamin intake from human milk fortifier, preterm formula, and parenteral nutrition could not be quantified completely on a day-to-day basis. The increase in 25-(OH)D and the associated clinical outcomes should therefore be interpreted as supportive findings for a combined supplementation strategy rather than definitive evidence of an independent vitamin D effect. Although ALP decreased to a greater extent in the combination group, calcium, phosphorus, and PTH did not differ significantly between groups. Because ALP is also influenced by growth, nutrition, and illness severity, these findings do not establish a uniform improvement in bone metabolism.

The observed changes in respiratory and inflammatory markers are biologically plausible. Caffeine may promote spontaneous breathing and weaning from ventilation, vitamin A may support alveolar epithelial integrity, and vitamin D may modulate inflammation through innate and adaptive immune pathways. Perinatal inflammatory biomarkers have been associated with later respiratory disease in preterm infants (25). However, only peripheral blood markers were measured, and only at two time points; local pulmonary inflammation, oxidative stress, and structural lung development were not assessed. The study therefore cannot establish that reduced inflammation mediated the association between combination therapy and BPD. Post-treatment PaO_2_ should also be interpreted in conjunction with FiO_2_, SpO_2_, and respiratory support status. Standardized arterial sampling, concurrent documentation of support parameters, and repeated analyses among infants who remained on respiratory support were implemented to reduce interpretive bias arising from differences in support status.

Several design and analytical features strengthened the robustness of the analysis. Exposure was fixed at a common 72 h landmark to reduce immortal time bias. All 126 eligible infants who survived to the landmark were included in the outcome cohort; deaths before 36 weeks were incorporated into the death-or-BPD composite. BPD severity was assessed using the Jensen criteria, and structured follow-up was used for infants discharged before 36 weeks. The timing of pre- and post-treatment testing and the conditions for arterial sampling were standardized. Linear mixed-effects models, multivariable regression, FDR correction, and IPTW were applied, and sensitivity analyses addressed infection, late-onset sepsis, and respiratory support status. Nevertheless, these approaches cannot replace randomization.

This study has several important limitations. First, the single-center retrospective design and non-randomized treatment allocation are susceptible to confounding by indication, clinician-level treatment preference, selection bias, and residual confounding. Although pre-landmark enteral route availability, feeding tolerance, contraindications, product availability, and clinical stability were extracted and explored, these variables formed part of the treatment selection process and could not fully reproduce the attending neonatologist's contemporaneous clinical judgment. Preliminary data availability screening and complete-case analyses may also have introduced selection bias because infants with incomplete exposure, covariate, outcome, or paired longitudinal data were excluded. The included infants may therefore differ systematically from the source population, and the characteristics of records excluded before detailed eligibility review could not be fully compared. Even after IPTW balanced the measured covariates, unrecorded SpO_2_ variability, fluid management, and other clinical decisions remained uncontrolled. Second, although the 72-h landmark design reduced immortal time bias, it excluded infants who died before the landmark; the findings therefore apply only to infants who survived to 72 h and cannot be generalized to all infants born at <32 weeks. Third, the study spanned 2022–2024, during which clinical protocols and drug availability may have changed. Admission period was included in the propensity score model, but residual temporal trends may remain. Fourth, the sample was limited, with only 31 BPD events and few grade 2–3 BPD, grade 3 BPD, and <25-week subgroup events; exploratory estimates may therefore be unstable. Fifth, although the propensity score model included minimal feeding and availability of human milk before the landmark, and the primary models did not adjust for potentially mediating post-treatment feeding milestones, vitamin A and D intake from fortifiers, preterm formula, and parenteral nutrition was not converted to cumulative daily doses. This leaves possible nutrition-related residual confounding. Sixth, because the combination product contained both vitamins, their independent contributions could not be separated, and the regimen cannot be considered equivalent to the classic high-dose intramuscular vitamin A regimen. Seventh, follow-up ended at 36 weeks’ PMA or discharge; long-term pulmonary function, rehospitalization, and neurodevelopmental outcomes were unavailable.

Future studies should use standardized caffeine, respiratory support, and nutritional pathways in prospective multicenter randomized trials or rigorously designed comparative studies. The dose, timing, route, and baseline status of vitamins A and D should be evaluated separately, with prespecified outcomes including death or BPD, BPD severity, and long-term respiratory and neurodevelopmental outcomes. Routine use of this combined strategy for BPD prevention should be considered only after replication in independent cohorts and confirmation in randomized studies.

## 5 Conclusion

In this single-center, 72-h landmark retrospective cohort, the early addition of vitamin A/D drops to caffeine citrate was associated with lower risks of BPD and death or BPD, more favorable short-term changes in respiratory and inflammatory markers, and shorter durations of respiratory support and hospitalization, without evidence of a serious safety signal. Findings for grade 2–3 BPD and ordinal BPD severity were exploratory. Given the non-randomized treatment allocation, landmark selection, limited sample size, and residual confounding, these findings do not establish that the combined regimen prevents BPD and require confirmation in prospective multicenter studies.

## Data Availability

The original contributions presented in the study are included in the article/Supplementary Material; further inquiries can be directed to the corresponding author.
