# Supplementary material for: Association of caffeine citrate plus vitamin A/D drops with bronchopulmonary dysplasia and respiratory outcomes in preterm infants born at <32 weeks’ gestation: a 72-h landmark retrospective cohort study
Source: Front Pediatr. 2026 Aug 26;14:1923440. doi: 10.3389/fped.2026.1923440 (PMC13553532; doi:10.3389/fped.2026.1923440)
Supplement: Supplementary file 1 [file Supplementaryfile1.docx]

**Association of Caffeine Citrate Plus Vitamin A/D Drops With Bronchopulmonary Dysplasia and Respiratory Outcomes in Preterm Infants Born at <32 Weeks' Gestation: A 72-Hour Landmark Retrospective Cohort Study**

Rongying Su¹, Yaohua Pei²*

**Supplementary Material**

**Supplementary Table S1. Changes in ALP, calcium, phosphorus, and PTH before and after treatment**

| **Variable** | **Caffeine, pre** | **Caffeine, post** | **Combination, pre** | **Combination, post** | **Adjusted difference in change (95% CI)** | **Raw P** | **FDR-adjusted P** |
| --- | --- | --- | --- | --- | --- | --- | --- |
| ALP (U/L) | 604.97±81.43 | 628.41±92.05 | 599.07±79.23 | 536.88±90.28 | −87.15 (−106.74–−67.55) | <0.001 | <0.001 |
| Calcium (mg/dL) | 9.58±0.57 | 9.62±0.61 | 9.55±0.56 | 9.69±0.54 | 0.11 (−0.02–0.23) | 0.107 | 0.178 |
| Phosphorus (mg/dL) | 5.95±0.67 | 6.07±0.71 | 5.79±0.63 | 5.85±0.69 | −0.07 (−0.22–0.08) | 0.365 | 0.456 |
| PTH (pg/mL) | 62.13±9.93 | 61.05±12.34 | 59.72±11.83 | 58.08±12.88 | −0.46 (−2.44–1.51) | 0.646 | 0.646 |

Note: 25-(OH)D, ALP, calcium, phosphorus, and PTH were treated as five outcomes within the same bone-metabolism domain and corrected together for multiple comparisons. 25-(OH)D is presented in Table 2. 25-(OH)D, 25-hydroxyvitamin D; ALP, alkaline phosphatase; PTH, parathyroid hormone; CI, confidence interval; FDR, false discovery rate.

**Supplementary Table S2. Covariate balance before and after IPTW**

| **Covariate** | **Absolute SMD before weighting** | **Absolute SMD after weighting** |
| --- | --- | --- |
| Gestational age | 0.172 | 0.033 |
| Birth weight | 0.138 | 0.041 |
| Grade III–IV RDS | 0.003 | 0.020 |
| PDA diagnosed ≤72 h | 0.095 | 0.031 |
| Chorioamnionitis | 0.059 | 0.025 |
| Initial FiO₂ | 0.131 | 0.030 |
| Initial MAP | 0.081 | 0.024 |
| Initial PaCO₂ | 0.090 | 0.019 |
| Initial invasive respiratory support | 0.067 | 0.022 |
| Admission in 2024 | 0.073 | 0.028 |
| Minimal enteral feeding within 24 h | 0.070 | 0.027 |
| Human milk available within 24 h | 0.077 | 0.032 |

Note: IPTW, inverse probability of treatment weighting; SMD, standardized mean difference; RDS, respiratory distress syndrome; PDA, patent ductus arteriosus; FiO₂, fraction of inspired oxygen; MAP, mean airway pressure; PaCO₂, arterial partial pressure of carbon dioxide. Only variables available before or at the 72-hour landmark were included in the propensity-score model.

**Supplementary Table S3. Descriptive outcomes among infants born at <25 weeks' gestation**

| **Variable** | **Caffeine group (n=8)** | **Combination group (n=6)** |
| --- | --- | --- |
| Death before 36 weeks PMA | 2 (25.0) | 1 (16.7) |
| BPD among survivors | 5/6 (83.3) | 3/5 (60.0) |
| Death or BPD | 7 (87.5) | 4 (66.7) |

Note: Because of the limited sample size and event counts in this subgroup, the results are descriptive only; no unstable regression estimates were calculated. BPD, bronchopulmonary dysplasia; PMA, postmenstrual age.

**Supplementary Table S4. Pre-landmark clinical factors related to initiation of vitamin A/D drops**

| **Variable** | **Caffeine group (n=60)** | **Combination group (n=66)** | **P value** | **Absolute SMD** |
| --- | --- | --- | --- | --- |
| Oral or gastric-tube route available by 72 h | 58 (96.7) | 65 (98.5) | 0.605 | 0.118 |
| Minimal enteral feeding initiated by 72 h | 52 (86.7) | 61 (92.4) | 0.382 | 0.188 |
| Pre-landmark feeding intolerance | 7 (11.7) | 4 (6.1) | 0.349 | 0.197 |
| Relevant baseline contraindication to vitamin A/D supplementation | 2 (3.3) | 0 | 0.225 | 0.260 |
| Vitamin A/D product available before 72 h | 57 (95.0) | 66 (100) | 0.105 | 0.320 |
| Clinical instability precluding enteral supplementation before 72 h | 6 (10.0) | 4 (6.1) | 0.517 | 0.145 |

Note: Data are presented as n (%). P values were calculated using Fisher exact tests. Absolute SMDs quantify allocation-related imbalance. Feeding intolerance was assessed from records obtained before the 72-hour landmark. Relevant contraindications included baseline biochemical abnormalities or documented clinical conditions considered incompatible with vitamin A/D supplementation. SMD, standardized mean difference.

**Supplementary Table S5. Diagnostics and outcome estimates from the expanded propensity-score weighting sensitivity analysis**

| **Measure** | **Expanded IPTW result** |
| --- | --- |
| Additional allocation-related covariates | Enteral-route availability by 72 h; pre-landmark feeding intolerance; documented product availability |
| Untruncated stabilized weight range | 0.49–3.08 |
| Truncated stabilized weight range (1st–99th percentiles) | 0.59–2.48 |
| Weighted effective sample size | 117.6 |
| Maximum post-weighting absolute SMD | 0.047 |
| BPD among survivors | Weighted OR 0.46 (95% CI, 0.20–1.06); P = 0.068 |
| Death or BPD in the landmark cohort | Weighted OR 0.43 (95% CI, 0.19–0.96); P = 0.040 |

Note: The expanded propensity-score model included all covariates from the primary IPTW model plus enteral-route availability by 72 hours, pre-landmark feeding intolerance, and documented product availability. Stabilized weights were truncated at the 1st and 99th percentiles. Weighted ORs and 95% CIs were estimated using robust sandwich standard errors. BPD, bronchopulmonary dysplasia; IPTW, inverse probability of treatment weighting; OR, odds ratio; SMD, standardized mean difference.
